# Hyponatremia Intervention Trial (HIT): Study Protocol of a Randomized, Controlled, Parallel-Group Trial With Blinded Outcome Assessment

**DOI:** 10.3389/fmed.2021.729545

**Published:** 2021-09-06

**Authors:** Julie Refardt, Anissa Pelouto, Laura Potasso, Ewout J. Hoorn, Mirjam Christ-Crain

**Affiliations:** ^1^Departments of Endocrinology, Diabetology and Metabolism University Hospital Basel, Basel, Switzerland; ^2^Department of Clinical Research, University of Basel, Basel, Switzerland; ^3^Clinical Trial Unit, Department of Clinical Research, University of Basel and University Hospital Basel, Basel, Switzerland; ^4^Department of Internal Medicine, Division of Nephrology and Transplantation, Erasmus MC, University Medical Center Rotterdam, Rotterdam, Netherlands

**Keywords:** hyponatremia, SIAD(H), mortality, outcome, trial, diuretics [MeSH], urea, sodium

## Abstract

**Background:** Hyponatremia is the most common electrolyte disorder with a prevalence of up to 30% in hospitalized patients. In contrast to acute hyponatremia where the need for immediate treatment is well-recognized, chronic hyponatremia is often considered not clinically relevant. This is illustrated by reports showing that appropriate laboratory tests are ordered in <50% of patients and that up to 75% are still hyponatremic at discharge. At the same time, emerging evidence suggests an association between hyponatremia and adverse events including increased risk of mortality and rehospitalization.

**Methods:** This is a randomized (1:1 ratio) controlled, superiority, parallel-group international multi-center trial with blinded outcome assessment. In total 2,278 participants will be enrolled. Participants will be randomly assigned to undergo either targeted correction of plasma sodium levels or standard of care during hospitalization. The primary outcome is the combined risk of death or re-hospitalization within 30 days.

**Discussion:** All data on hyponatremia and mortality are derived from observational studies and often lack methodologic robustness. Consequently, the direct impact of hyponatremia on mortality and rehospitalization risk is still debated, resulting in a clinical equipoise whether in-hospital chronic hyponatremia should be treated or not. Therefore, a randomized controlled trial is required to study whether targeted plasma sodium correction reduces the risk of mortality and rehospitalization associated with hyponatremia.

**Clinical Trial Registration:**www.ClinicalTrials.gov, identifier: NCT03557957.

## Background

Hyponatremia is the most common electrolyte disorder with a prevalence of up to 30% in hospitalized patients (1). In contrast to acute hyponatremia where the need for immediate treatment is well-recognized due to severe clinical symptoms caused by cerebral edema (2, 3), chronic hyponatremia is often considered not clinically relevant (4). A registry analysis from 225 sites in the European Union and the United States showed that appropriate laboratory tests to evaluate the etiology of hyponatremia were obtained in <50% of patients and up to 75% of patients was still hyponatremic at discharge (5).

However, there is increasing evidence showing an association between chronic hyponatremia and clinical complications including gait alterations, attention deficits, falls (6), bone loss, and fractures (7). Furthermore, hyponatremia is associated with worse prognosis in patients with heart failure (8–10), myocardial infarction (11, 12), liver cirrhosis (13), pneumonia (14), pulmonary embolism (15), chronic kidney disease (16) and in patients admitted to the intensive care unit (17). The increased risk of mortality that arises from several observational studies might result from an intricate combination of factors, including the underlying condition and concomitant diseases (18–20). Whether these associations indicate a causal relationship has not been established yet. The debate on whether hyponatremia contributes directly to mortality or is rather a surrogate marker for the severity of the underlying condition is ongoing (21).

Several studies have shown differences in neurocognitive and muscular function (6, 22), as well as an improvement in quality of life (23) and a reduction in costs through hyponatremia treatment (24). The key question, however,—whether targeted correction of hyponatremia reduces the risk of mortality and rehospitalization—has not been addressed. All data regarding hyponatremia and mortality originate from observational studies or retrospective analyses (25–27). A meta-analysis of fifteen observational studies with a total of 13,816 patients reported that any improvement of hyponatremia was associated with a reduced risk of overall mortality (OR 0.57; 95% CI 0.40–0.81) (26). Randomized controlled trials studying the effect of hyponatremia treatment on mortality were limited to studies assessing the effect of one drug class only: vaptans. Furthermore, inclusion was restricted to patients with either hyper- or euvolemic hyponatremia. Treatment with tolvaptan corrected hyponatremia, yet no difference in 30-day mortality between tolvaptan and placebo was found (28). Other small randomized controlled trials (*n* = 20–60) with vaptans reported similar outcomes (29–31). However, the results from these studies cannot be generalized to other treatment forms of hyponatremia, as vaptans bear the risk of overcorrection (32), which in turn can have serious neurologic ramifications including osmotic demyelination syndrome (33–35). Hyponatremia is a heterogeneous and complex disorder which may be best managed through a tailored approach with individualized treatment, rather than by applying a single treatment modality.

Except for the vaptan-trials, which cannot be extrapolated to the heterogeneous in-hospital hyponatremic population, there is a complete lack of randomized controlled trials investigating whether treatment of hyponatremia reduces the risk of mortality and rehospitalization associated with hyponatremia. To address this question, the aim of this international multi-center randomized controlled trial is to determine the benefits and harms of targeted hyponatremia correction on the combined endpoint 30-day mortality and rehospitalization rate.

## Methods

### Study Design

HIT is a randomized (1:1 ratio) controlled, superiority, parallel-group international multi-center study with blinded outcome assessment.

- 2,278 hospitalized patients with hypotonic hyponatremia <130 mmol/l will be included
- Participants will be randomly assigned to undergo either targeted correction of hyponatremia in addition to current standard care or current standard care during the course of the index hospitalization
- The primary outcome is assessed 30 days after inclusion
- Outcome assessment at 30 days and 1 year will be blinded to treatment allocation.

**Figure 1:**
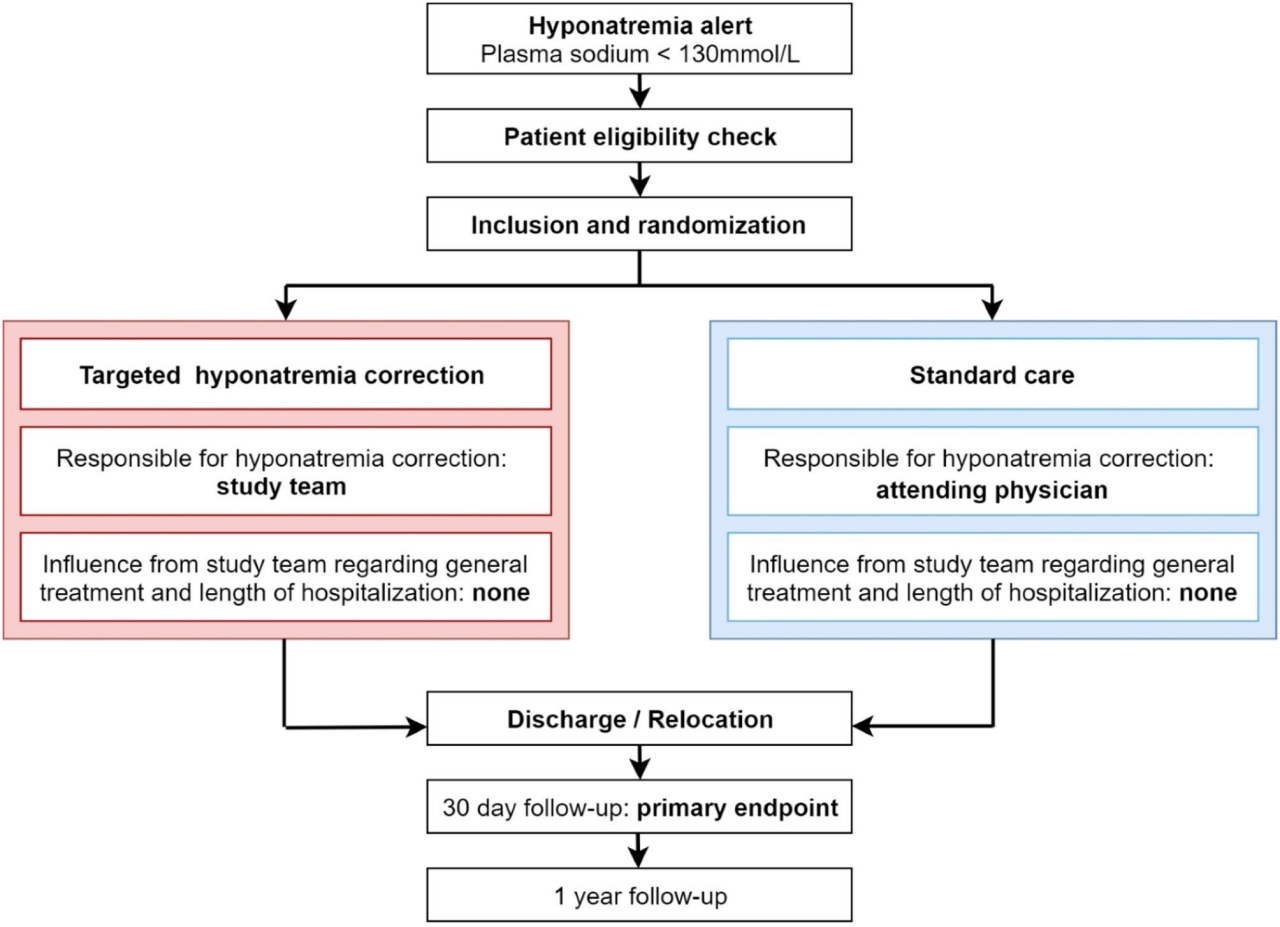
Study design flowchart.

The study design flowchart is depicted in Figure 1.

A detailed overview of study procedures and assessments during index hospitalization, at 30 days and 1 year is included in Supplementary Table 1.

### Eligibility Criteria

See Table 1 for the inclusion and exclusion criteria.

**Table 1:** Inclusion and exclusion criteria.

**Inclusion criteria**	**Exclusion criteria**
All adult hospitalized patients with hypotonic hyponatremia <130 mmol/l	Severe symptomatic hyponatremia in need of intensive care treatment and/or of acute correction with 3% saline
Informed Consent as documented by signature	Non-hypotonic hyponatremia with plasma osmolality >300 mOsm/kg or in absence of plasma osmolality but with any circumstances suggestive of non-hypotonic hyponatremia End of life care
	Kidney failure requiring dialysis
	Acute liver failure
	Wernicke encephalopathy
	Hepatic encephalopathy during the last 2 months
	Hepatorenal syndrome
	Patients in the isolation ward due to hematological diseases
	Pregnancy/breastfeeding

### Screening and Recruitment

To enable a daily screening of all hospitalized patients, plasma sodium values ordered through regular care will be screened using an electronic alert system by the local study physician.

Patients will be contacted as soon as the diagnosis of hypotonic hyponatremia <130 mmol/l is established. Patients may be eligible for study participation at any time during their hospitalization.

### Randomization

Participants will be randomly 1:1 assigned to either the targeted-sodium-correction group or the standard treatment group. Randomization will be performed by the online database system SecuTrial®, stratified by study site and using permuted block of random sizes. The allocation sequence will be implemented by the study data manager of the clinical trial unit Basel, Switzerland. Randomization will be done directly after inclusion of each patient in the online database system.

### Blinding

Attempts to blind the hyponatremia interventions would create an artificial care environment with a substantial deviation from routine care. To minimize the risk of bias for the outcome assessment, outcome assessors are not involved in the study and are not aware of the treatment allocation. All outcome data will be entered into a separate database with no link to other study data.

### Study Intervention

#### A. Intervention Group

The recommended algorithm for diagnosing and treating hyponatremia is based on the current *European Clinical Practice Guidelines (ECPG)(2)* and the *Expert Panel Recommendations (EPR)(3)*. The study group will check for guideline updates regularly during the course of the study and if applicable amend the algorithm accordingly. The local study task force will be trained in the recommended diagnostic classification and treatment procedure prior to start of the study.

1. **Diagnosis:**
  Diagnosis of hyponatremia will be made by the local study physician and will be reviewed by the local principal investigator. According to the *ECPG*, patients will be classified into the 3 main groups of hypotonic hyponatremia (decreased/normal/increased extracellular fluid volume; details see Supplementary Figure 1). If the diagnosis is uncertain, the most likely categorization will be chosen. This initial diagnosis can and shall be changed according to treatment response and/or additional clinical information. The final diagnosis will be made before the patient is discharged.
2. **Treatment:**
  The proposed treatment recommendation will be based on the predefined algorithms which were developed in accordance to the ECPG(2) and the EPR(3) (details see Supplementary Figure 2). The treatment algorithm is a recommendation only, meaning the proposed algorithm is intended as a guideline rather than a strict treatment plan. This approach takes into account the sometimes limited data for certain treatments (36) and enables the local expert to decide upon the best treatment for every patient individually based on the available treatment options. Given that the hyponatremia patient population in clinical practice is very heterogeneous, we decided a tailored approach for hyponatremia correction would be in the best interest of the patients. Except for correction of hyponatremia, the local study team will not influence other treatment goals and decisions, which will be fully at the discretion of the attending physicians. Treatment response and adherence will be evaluated daily. The study nurse will report to the study physician, who will additionally examine the patient if clinically indicated. The study physician will then—under the supervision of the local principal investigator—adapt treatment recommendation according to the predefined treatment goals and inform the treating physicians about the treatment modification. The general treatment goal is to raise plasma sodium levels by at least 2 mmol/l/24 h. These treatment goals also define the time until the next treatment step should be implemented. The following management rules apply:
  - increase ≥2 mmol/l/24 h and <12 mmol/l/24 h and 18 mmol/l/48 h: current treatment option will be maintained until plasma sodium level has normalized
  - increase <2 mmol/l/24 h: treatment will be intensified or switched to a different modality
  - increase >12 mmol/l/24 h and/or 18 mmol/l/48 h: treatment will be discontinued and, if needed, counter regulatory actions (i.e., hypotonic infusion, administration of desmopressin) will be administered according to the decision of the local principal investigator.

Episodes of overcorrection (defined as increase >12 mmol/l/24 h, respectively 18 mmol/l /48 h) and their complications will be recorded. Targeted plasma sodium correction will be maintained—for a maximum of 30 days—until normonatremia is achieved or when the patient is discharged from hospital. At discharge the last available plasma sodium level and whether or not patient was treated according to protocol will be recorded. Not treated according to protocol applies in the case treatment recommendation is not implemented or the patient does not adhere to the prescribed treatment (details see below “Compliance with study intervention”). In case of hospitalization exceeding 30 days, the last plasma sodium level before stopping the intervention (day 31) will be recorded as the discharge plasma sodium. Treatment after discharge will not be influenced by the study team, however a treatment recommendation and request to monitor plasma sodium levels will be given to the general practitioner in the discharge letter.

#### B. Control Group (Standard Care)

##### 1. Diagnosis

Laboratory analysis (blood and urine samples) to diagnose hyponatremia will be at the discretion of the attending physician according to local standards of care. The final hyponatremia diagnosis will be made by the study team at discharge according to all available clinical and laboratory information as well as treatment response.

##### 2. Treatment

Treatment of hyponatremia will be solely at the discretion of the attending physicians. The study team will not intervene with the treatment in any way. Treatment decisions as well as the course of the plasma sodium level will be recorded after patient is discharged from hospital using the medical records and patient charts. To ensure the planned outcome assessment, it will be generally recommended to measure plasma sodium levels 3 times weekly or more frequently if clinically indicated, before discharge and after 30 days.

##### Compliance With Study Intervention in Both Study Groups

Adherence will be evaluated as follows:

(A) Intervention group:

- Administration of the prescribed treatment will be monitored daily through visits on the hospital ward by the local study nurse.
- Treatment response will be monitored daily for a maximum of 30 days by the study physician and treatment will be adjusted accordingly.

(B) Control group (standard care):

- Diagnostic evaluations, plasma sodium levels and prescribed treatment will be recorded by the local study nurse through evaluation of the patient-chart and laboratory values after patient is discharged from hospital.
- The study team will not contact or advise the attending physicians in any way.

### Follow-Up

All patients (or if they cannot be reached their general practitioner) will be contacted 31 days and 1 year after the initial hospitalization by a member of the local study team who was not involved during the hospitalization and who is blinded to the allocated treatment group.

The following will be evaluated in a standardized survey:

**Primary outcomes:**

- Survival status, date of death (if applicable)
- Rehospitalization since index hospitalization

**Secondary outcomes:**

- Number of falls within 30 days since randomization
- Occurrence of any medically confirmed fractures since index hospitalization
- Recurrence rate of hyponatremia since index hospitalization. Additional data from the general practitioner or medical records will be checked to further validate this point, if indicated
- Complications due to overcorrection of hyponatremia (i.e., osmotic demyelination according to MRI scan or neurological diagnosis)
- Mentioning of hyponatremia and its etiology in the discharge letter from the index hospitalization (30 day follow up only)
- Score of quality of life EQ-5D-5L Test (if applicable).

### Objectives and Outcomes

#### Objectives

The primary objective is to investigate whether in hospitalized hyponatremic patients targeted correction of plasma sodium concentration plus standard care compared to standard care alone reduces the combined 30-day risk of mortality or rehospitalization. The secondary objective is to investigate whether the intervention has favorable effects on other patient relevant outcomes. Lastly, the study aims to assess the tolerability and risks of targeted correction of plasma sodium concentration.

See Supplementary Table 2 for the complete set of outcomes.

### Data Entry and Quality Control

Study data are captured in an online Electronic Data Capture system based at the IT-Department of the University Hospital Basel (SecuTrial® database). Data entered into the electronic case report form is validated for completeness and discrepancies automatically. An audit trail system maintains a record of initial entries and changes (reasons for changes, time and date of changes, user identification of entry and changes). Monitoring visits will regularly take place at the investigator's site during the course of the study. To this purpose, source data/documents are made accessible to monitors and questions are answered during monitoring.

### Sample Size Calculations and Statistical Analyses

#### Determination of Sample Size

Sample size was estimated based on our already completed observational multi-center study in 298 patients with profound hyponatremia (37, 38). The study showed a 30-day mortality rate of 6.4% and a 30-day rehospitalization rate of 18.5%. Of the re-hospitalized patients, ~2% died and thus were counted twice. We therefore propose a combined event rate of 23% under standard care. Our systematic review identified only one study that investigated the impact of targeted correction of hyponatremia on mortality and rehospitalization (25). In this observational study, an absolute reduction of the combined event rate of 7.1% (33.7 vs. 26.6%) was reported. Through our intervention, we expect to achieve an absolute risk reduction for mortality and rehospitalization of 5 %. Sample size was calculated for a Pearson's χ^2^-test. We set 80% power for a two- sided test with an α error of 5%. A drop-out rate of 10% was assumed. In order to have 2050 evaluable patients, 2,278 patients must be recruited.

#### Datasets to Be Analyzed

The full analysis set will contain all patients in whom a treatment was randomly allocated and who received the allocated treatment for at least 24 h or who died within the first 24 h. Patients will be analyzed according to treatment allocation.

The per-protocol set will contain all patients who fulfilled following criteria: (1) all patients for whom the primary outcome is available, (2) all patients who received treatment according to protocol, meaning that patients in whom the intervention was stopped early (e.g., due to drop-out, start of best supportive care) or in whom intervention was not executed according to the recommendation of the study team (e.g., treating physician refusing intervention) will be excluded from the analysis.

#### Primary Analysis

Analysis of the primary outcome will follow the intention-to-treat principle. It will be based on the full analysis set. To test whether the study intervention differs from standard care 30 days after randomization, the Pearson's χ^2^-test without Yates's correction will be applied. Threshold for rejecting the null hypothesis will be α = 5%. The estimated absolute difference between study arms will be reported alongside its 95% confidence interval.

#### Secondary Analysis

Each of the components of the primary outcome, death and rehospitalization, will be analyzed separately as described for the primary outcome. Time to death and rehospitalization after 30 days and 1 year will be analyzed by fitting Cox proportional hazards regression models with trial arm as explanatory variable. Hazard ratios including their confidence intervals will be reported. Patients will be censored on their last known follow-up. To illustrate the course of deaths and re-hospitalizations in the two treatment arms, a Kaplan-Meier Curve will be presented.

Length of hospital stay will be calculated in days considering index and each rehospitalization separately and by the sum of all hospital stays. Standard summary statistics for numerical data will be performed. If distribution of data allows, significant differences of mean of length of hospital stay between arms will be assessed with a *t*-test (two-sided α = 5%). If distribution is skewed, data will be transformed or other models selected.

The secondary outcomes (falls, fractures, course of hyponatremia, recurrence of hyponatremia, severe symptomatic hyponatremia, overcorrection of hyponatremia) will be analyzed as described for the primary outcome. To account for multiple occurrences of falls, fractures, and recurrence of hyponatremia after 1 year, we will apply a Poisson model.

Diagnostic performance of biomarkers will be assessed by calculating sensitivity, specificity and accuracy as well as best cut-offs. Gold standard is the correct diagnosis of etiology of hyponatremia, which is expert opinion based on biochemistry results and treatment response.

Analyses of differences of quality of life score and of executive functioning score between arms will be performed also considering grade of hyponatremia and its correction. Multivariate linear models will be used to explore possible associations.

### Safety Analysis

Incidence rates of AE/SAE, including symptomatic hyponatremia and plasma sodium overcorrection during index hospitalization, will be calculated at study end as described for the primary outcome. Summary statistics of multiple occurrences of AE/SAE within individual patients, will be presented separately. An independent data safety monitoring board will oversee the trial and conduct a safety-analysis after 1,000 patients have completed the 30-day follow-up.

### Subgroup Analyses

We are interested in whether the treatment effect, i.e., the difference between the two treatment arms, on the primary outcome and its components varies between the following patient subgroups: sex (female and male), age (<70 and ≥ 70 years), severity of hyponatremia (mild-moderate: 120–130 mmol/l and severe: <120 mmol/l), underlying disease (stroke, heart failure, SIAD, hepatic impairment, chronic kidney disease, malignancy, surgical diagnosis) as well as the main treatment used (fluid restriction, diuretics, infusion, urea, vaptans). To this end, we will fit logistic regression models, including treatment arm, subgroup and the interaction term as explanatory variables. Significance for interaction is defined by α = 5%. If a significant interaction effect results, the treatment effect will be calculated for the respective outcome and subgroups separately.

## Discussion

The current study protocol presents the design for a randomized controlled trial to study whether targeted treatment of hyponatremia reduces the risk of mortality and rehospitalization associated with hyponatremia. This trial is conducted in different European hospitals, including non-academic centers ensuring generalizable data is produced. In addition, adult patients with hypovolemic, euvolemic and hypervolemic hyponatremia will be included allowing for a broad representation of hyponatremic patients.

Assessing the contributory effect of hyponatremia on mortality is difficult (21), as patients presenting with hyponatremia often have severe and complex underlying conditions (39). A recent large retrospective study (*n* = 94,352) attempted to minimize the influence of confounding by optimizing comparability through propensity-score matching of hyponatremic with non-hyponatremic patients (40). Hyponatremia was indeed associated with increased risk of short-term adverse events including in-hospital mortality. Interestingly, the increased risk of in-hospital mortality did not apply to patients with hyponatremia secondary to SIAD. In a previous study designed to evaluate mortality risk specifically associated with SIAD, mortality rates were lower in patients with SIAD compared to patients with hyper- or hypovolemic hyponatremia. However, mortality rates in SIAD were still higher compared with normonatriemic patients (41).

Another study analyzed mortality rates separately for malignant and non-malignant SIAD and found that only malignant SIAD was associated with higher risk of mortality (38). Especially drug-induced SIAD predisposed to pronounced hyponatremia (<120 mmol/l), yet the mortality rate in this group was not increased (38). Paradoxically, mortality rates have repeatedly been reported lower in patients with more severe hyponatremia (18, 20, 42, 43). An explanation may be that patients with milder hyponatremia are more often admitted because of a severe underlying disease, whereas patients with severe hyponatremia are more often admitted because of hyponatremia (44). Another explanation may be a more active therapeutic approach in severe hyponatremia compared to moderate and mild hyponatremia (5, 38, 41). Indeed, a recent single-center study showed that over a 10-year period active intervention and specialist management of severe hyponatremia have increased. The improved management of severe hyponatremia was, in turn, associated with a decline in mortality (45).

A number of pathophysiological mechanisms have been proposed to explain how hyponatremia could potentially lead to increased mortality risks. It is, however, important to stress that thus far these are mainly theories, since a causal relationship between hyponatremia and increased mortality risk has not been established yet. Below we will discuss four proposed mechanisms leading to the increased mortality risks.

First, hyponatremia could contribute to mortality through concomitant hypoxia (i.e., respiratory insufficiency). The concurrence of hyponatremia and hypoxia is strongly associated with mortality in human (46, 47) and animal studies (48), possibly because hypoxia interferes with the compensatory mechanisms that are normally responsible for brain adaptation to hyponatremia (49). Second, adaptations of cardiomyocytes to chronic hyponatremia could negatively impact cardiac function and the patient's survival (50). When challenged with extracellular hypotonicity, swelling of cardiomyocytes is prevented through efflux of taurine (51, 52)—one of the most prevalent organic osmolytes in cardiomyocytes. Taurine, however, is considered essential for maintaining normal contractile function (53). Therefore, taurine depletion is linked to cardiomyopathy. Indeed, taurine depletion in animal models led to cardiomyopathy, which was reversible after taurine repletion (54, 55). Third, chronic hyponatremia can contribute to mortality by promoting or accelerating cellular senescence. Chronic hyponatremia in animal models induced multiple pathologies commonly associated with aging, including bone loss, hypogonadism, sarcopenia and cardiomyopathy (56). If hyponatremia induces cellular senescence this might explain why multiple organ systems are affected and the overall mortality risk is increased. Finally, osmotic stress could increase mortality risk in patients with advanced solid tumors, as hyponatremia in this population was found to be an independent predictor for poor prognosis (57). Osmotic stress may induce the transcription of serum and glucocorticoid-regulated kinase 1 (SGK1) gene (58). Increased SGK1 expression subsequently inhibits cell apoptosis and stimulates cell growth and proliferation (58, 59), ultimately leading to increased risk of metastases in patients with solid tumors (57).

In the absence of randomized controlled trials, the potential impact of hyponatremia on mortality remains unresolved. Therefore, randomized controlled trials are required to further the field.

Even though randomized controlled trials are considered the best study design to assess causality, some limitations of our trial should be mentioned. First, this trial takes place in a real-world setting, so logistical obstacles and reduced adherence to recommended treatment may occur. Non-compliance by both the treating physician and/or the patient may compromise the number of patients in the per protocol analysis. Also, full hyponatremia correction may not be achieved in patients who are quickly discharged. More specifically, we take into account that both premature discharge and non-compliance will affect the number of patients in which full hyponatremia correction can be realized. As a consequence, in a number of patients plasma sodium concentration is expected to increase but not reach normal levels within the hospitalization period. Early discharge can especially limit the chance of successful hyponatremia correction in patients who require treatment escalation or sequential use of various treatment options. Yet, results from this trial will be a good reflection of everyday clinical practice. Second, we anticipate that this trial may increase the overall awareness for hyponatremia in the study centers which may lead to improved hyponatremia management in the control group. However, the problem of contamination is not expected to be significant enough to warrant a cluster-randomization design. Also, cluster-randomization designs are much more susceptible to selection bias and therefore do not outweigh the advantages of individual randomization (60). Third, to avoid contamination, the study team will not order diagnostic tests in the control group. As a result, the etiology of hyponatremia in the control group will be derived from the combination of all available clinical and laboratory findings and treatment response. Follow-up plasma sodium measurements in the control group will be recommended because this is necessary to ensure the planned outcome assessment. We acknowledge that this creates an area of tension between possibly contaminating standard of care and complete data collection to address the primary research question.

In summary, there is an equipoise whether in-hospital chronic hyponatremia should be treated or not. This study will be the first randomized controlled trial evaluating the effect of in-hospital hyponatremia correction on patient relevant endpoints. We expect the results of this trial to influence future hyponatremia management regardless of its outcome: if the intervention results in a significant reduction of mortality and rehospitalization rate, hyponatremia awareness and its consecutive treatment during hospitalization will change substantially. If the intervention, however, shows no effect on mortality and rehospitalization rate, hyponatremia will be recognized more as a marker of the severity of the disease and not as its cause.

## Ethics Statement

The study protocol was reviewed and approved by the Competent Ethics Committee of each participating center: University Hospital Basel, Basel, Switzerland; Kantonsspital Aarau, Aarau, Switzerland; Erasmus Medical Center Rotterdam, Rotterdam, The Netherlands; Kantonspital St. Gallen, St. Gallen, Switzerland; Bürgerspital Solothurn, Solothurn, Switzerland; Kantonsspital Liestal, Liestal, Switzerland; University Hospital Center Zagreb, Croatia; Universitätsklinikum Köln, Cologne, Germany; University Hospital Firenze, Florence, Italy. The patients provided their written informed consent to participate in this study.

## Author Contributions

JR and MC-C designed the study. JR, LP, EH, and MC-C wrote the study protocol. AP wrote the initial draft of this article. JR, MC-C, and EH revised the manuscript. All authors contributed to the article and approved the submitted version.

## Trial Status

Recruitment for this trial is ongoing. At the time of writing this manuscript 750 participants have been included in the trial.

## Hit Study Group

• ^**1**^**University Hospital Basel, Switzerland**

Prof. Mirjam Christ-Crain^1^, Julie Refardt^1^, Laura Potasso^1^, Deborah Vogt^1^, Brida Caviezel^1^, Sophie Monnerat^1^, Gabriela Bucklar^1^, Lara Gut^1^, Cornelia Imber^1^, Milica Popovic^1^, Susan Felder^1^, Tanja Vukajlovic^1^, Odile Christin Gaisl^1^, PD Lars G. Hemkens^1^, Prof. Stefano Bassetti^1^, Prof. Tobias Breidthardt^1^, Prof. Otmar Pfister^1^, Prof. Christian Müller^1^

• ^**2**^**Medical University Clinic Kantonsspital Aarau, Switzerland**

Prof. Beat Müller^2^, Prof. Philipp Schuetz^2^, Claudia Gregoriano^2^, Claudine Blum^2^, Alexander Kutz^2^

• ^**3**^**Erasmus MC, University Medical Center Rotterdam, The Netherlands**

Prof. Ewout Hoorn^3^, Adrienne Zandbergen^3^, Anissa Pelouto^3^

• ^**4**^**Kantonsspital Baselland, Switzerland**

Prof. Jörg Leuppi^4^, Cedrine Küng^4^, Andrea Roth^4^, Dominik Schnyder^4^

• ^**5**^**Bürgerspital Solothurn, Switzerland**

Gregor Lindner^5^, Basil Ryser^5^

• ^**6**^**Kantonsspital St. Gallen, Switzerland**

Stefan Bilz^6^, Prof. Michael Brändle^6^, Martina Bontognali^6^

• ^**7**^**University Hospital Zagreb, Croatia**

Darko Kaštelan^7^, Dora Pupovac^7^, Lana Šambula^7^

• ^**8**^**University Hospital Cologne, Germany**

Prof. Dr. Volker Burst^8^, Polina Todorova^8^, Sadrija Cukoski^8^

• ^**9**^**Careggi University Hospital Firenze, Italy**

Prof. Alessandro Peri, Dario Norello MD, Benedetta Fibbi MD, Cecilia Anceschi PhD, Giada Marroncini PhD, Laura Naldi PhD

## Conflict of Interest

The authors declare that the research was conducted in the absence of any commercial or financial relationships that could be construed as a potential conflict of interest.

## Publisher's Note

All claims expressed in this article are solely those of the authors and do not necessarily represent those of their affiliated organizations, or those of the publisher, the editors and the reviewers. Any product that may be evaluated in this article, or claim that may be made by its manufacturer, is not guaranteed or endorsed by the publisher.
