# Supplementary material for: Hyponatremia Intervention Trial (HIT): Study Protocol of a Randomized, Controlled, Parallel-Group Trial With Blinded Outcome Assessment
Source: Front Med (Lausanne). 2021 Sep 6;8:729545. doi: 10.3389/fmed.2021.729545 (PMC8450416; doi:10.3389/fmed.2021.729545)
Supplement: Supplementary file 1 [file Data_Sheet_1.PDF]

Table S1. Study procedures and assessments during index hospitalization

	DAYS IN TRIAL									
	Enroll-ment	**Randomi-zation**	Postallocation						**Follow up 1**	Follow up 2
Time point<br>Days in trial	$-t_0$	$\mathbf{t_0}$	t_1	tx_2	tx_3	tx_4	t_{xy}	Discharge	$\mathbf{t_{final1}}$<br>**30**	t_{final2}<br>365
**Trial participation**										
Eligibility screen	X									
Informed consent	X									
EQ-5D-5L test									X	X
Trail making test A	X							X		
Trail making test B								X		
Treatment allocation		X								
Diagnosis according to algorithm		X	(X)	(X)	(X)	(X)	(X)	X		
Treatment according to algorithm		X	X	X	X	X	X			
**Clinical assessment**										
Medical history	X								X	X
Physical exa-mination	X									
Evaluation adverse events / serious adverse events			X	X	X	X	X	X	X	X
Concomitant care review	X		X	X	X	X	X	X		
Outcomes and healthcare services utilization review (telephone interview)									X	X
**Laboratory assessment**										

Blood / urinary chemistry	X		(X)	(X)	(X)	(X)	(X)	X	X	X
Urinary chemistry	X									
Blood samples for biomarker measurement	X									

Postallocation measurements only applicable for intervention group. Parameters for control group will be assessed after discharge from index hospitalization.

(X) = only if applicable: e.g. evaluation of treatment response/adjustment of diagnosis

tx2-y = repeated daily until discharge of hospital (maximum 30 days)

final 1 = 30day follow up – blinded to treatment allocation

final 2 = 1 year follow up – blinded to treatment allocation
