# Supplementary material for: Hyponatremia Intervention Trial (HIT): Study Protocol of a Randomized, Controlled, Parallel-Group Trial With Blinded Outcome Assessment
Source: Front Med (Lausanne). 2021 Sep 6;8:729545. doi: 10.3389/fmed.2021.729545 (PMC8450416; doi:10.3389/fmed.2021.729545)
Supplement: Supplementary file 2 [file Data_Sheet_2.PDF]

Table S2. Primary, secondary and safety outcomes

**Primary outcome**	Combined risk of mortality and rehospitalization within 30 days
**Secondary outcomes**	Mortality within 30 days and 1 year for all patients as well as the predefined subgroups: patients with stroke, heart failure, SIAD, chronic hepatic impairment, chronic kidney disease, malignancies and surgical diagnosis.
	Rehospitalization within 30 days and 1 year for all patients as well as the predefined subgroups: patients with stroke, heart failure, SIAD, chronic hepatic impairment, renal impairment, malignancies and surgical diagnosis.
	Time to death and time to first rehospitalization in days
	Sodium normalization rate at discharge and time to normalization in days
	Presence of at least one normonatremic value during hospitalization, as a binomial variable
	Number of patients who reached a serum sodium level ≥130 mmol/l at discharge
	Change in plasma sodium level from study inclusion until discharge, maximum sodium change from baseline during index hospitalization and course of plasma sodium level during the hospitalization.
	Plasma sodium area under the curve (AUC) from study inclusion to day 5 and day 7 of hospitalization and normonatremic plasma sodium AUC from study inclusion to day 5 and day 7 of hospitalization
	Length of index hospitalization in days and days spent in normonatremia (in relation to length of index hospitalization)
	Number of falls within 30 days and number of fractures within 30 days and 1 year
	Rate of mentioning hyponatremia and its cause as a diagnostic point in the discharge letter (assessed at the 30 day follow up visit)
	Hyponatremia recurrence within 30 days and 1 year
	Diagnostic accuracy of biomarkers of fluid disorders (copeptin, MR-proANP, NT-proBNP, aldosterone, renin)
	Score of quality of life test EQ-5D-5L at day 31 and after 1 year
	Score of trail making test A on admission and at discharge as well as score of trail making test B at discharge
**Safety outcomes**	
	Adverse events/ serious adverse events* during intervention and follow-up
	Plasma sodium overcorrection during index hospitalization
	Adverse events due to plasma sodium overcorrection (assessed as neurological diagnoses, MRI)
	Severely symptomatic hyponatremia requiring intensive care treatment during index

	hospitalization

* Any unfavorable change in the health of a patient including abnormal laboratory findings of category III or more according to Common Terminology Criteria for Adverse Events (CTCAE) v4.0 will be recorded(60).
