# Supplementary figures and images for: Hyponatremia Intervention Trial (HIT): Study Protocol of a Randomized, Controlled, Parallel-Group Trial With Blinded Outcome Assessment
Source: Front Med (Lausanne). 2021 Sep 6;8:729545. doi: 10.3389/fmed.2021.729545 (PMC8450416; doi:10.3389/fmed.2021.729545)

Figure S1. Diagnostic algorithm

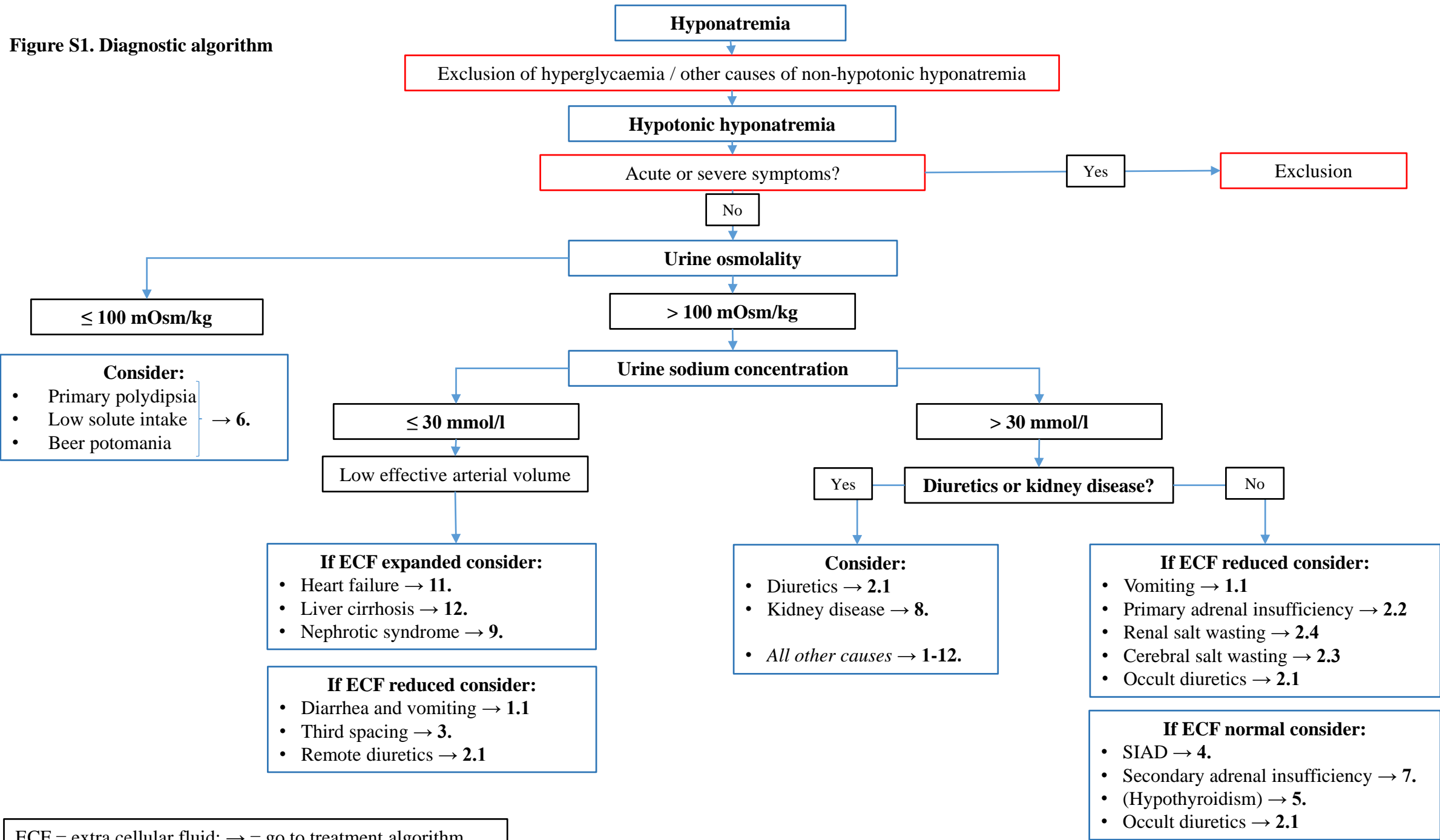

Supplement: Supplementary file 3 [file Data_Sheet_3.PDF]
