# Supplementary material for: Hyponatremia Intervention Trial (HIT): Study Protocol of a Randomized, Controlled, Parallel-Group Trial With Blinded Outcome Assessment
Source: Front Med (Lausanne). 2021 Sep 6;8:729545. doi: 10.3389/fmed.2021.729545 (PMC8450416; doi:10.3389/fmed.2021.729545)
Supplement: Supplementary file 4 [file Data_Sheet_4.PDF]

Figure S2. Treatment algorithm

Treatment options from different categories can be combined, no specific order required

REDUCED ECF

1. Non-renal sodium loss

1.1 Gastrointestinal / transdermal sodium loss

A: Infusion of balanced crystalloid solution 0.5-1.0 ml/kg per h
Increase / decrease according to clinical presentation & comorbidities
(e.g. caution in patients with chronic heart failure)

CAVE: sudden increase in urine output >100 ml/h signals risk sodium overcorrection

2. Renal sodium loss

2.1 Diuretics

A: Stop /reduce triggering medication

B: Infusion of balanced crystalloid solution according to 1.1

2.2 Primary adrenal insufficiency

A: Hydrocortisone 100mg i.v., followed by 50mg i.v. every 6-8 hours according to stress level

B: Additional fluid substitution (see 1.1) as needed

C: Evaluate additional administration of fludrocortisone 0.1 mg/d

2.3 Cerebral salt wasting syndrome

A: Balanced crystalloid solution 0.5-1.0 ml/kg per h, adjust according to clinical course

B: Evaluate additional administration of salt tablets 1-3/d

C: Evaluate additional administration of fludrocortisone 0.1 mg/d

CAVE: in case of active intracranial pathology (eg. recent intracranial surgery or SAH), intensive care treatment with 3% saline solution might be required

2.4 Salt loosing nephropathy

A: Stop /reduce triggering medication

B: Infusion of balanced crystalloid solution according to 1.1 as needed

3. Third spacing (Pancreatitis, sepsis, trauma, etc)

A: Balanced crystalloid solution 5 to 10 mL/kg per h
Increase / decrease according to clinical presentation & comorbidities
(e.g. caution in patients with chronic heart failure)

NORMAL ECF

4. Syndrome of inappropriate antidiuresis (SIAD)

(prior exclusion hypocortisolism / hypothyroidism for diagnosis mandatory)

A: Stop triggering medication if possible / treatment of underlying disease

B: Fluid restriction <0.5-1 L/d if:

- Urine-osmolality <500 mOsm/kg and/or Urine Na^++K^+< Plasma Na^+
- Absence of acute neurological trauma

C: Urea p.o. with/without combination fluid restriction (**B**):

- Start with 15-30 g/d, increase to 2 x 30 g/d as needed

CAVE: Contraindicated if patient is hyperuremic; Regular controls p-urea mandatory

D: Evaluate additional administration NaCl Tablets 2-3g/d and/or Furosemide 20mg 1-2x/d

E: Vasopressin receptor antagonists

- Start with 7.5-15 mg Tolvaptan/d, increase as needed

CAVE: risk of sodium overcorrection. Check water balance / electrolytes every 2-6 hours

5. Hypothyroidism

A: Start thyroxin substitution 1.5ug/kg bodyweight

CAVE: Evaluate ¼ - ½ starting dosage in patients >65 years or with known cardiovascular disease
Exclusion hypocortisolism before start substitution mandatory

6. High water, low salt intake

A: Fluid restriction <1 L/d

B: Additional high salt diet >6 g/d as needed

CAVE: Risk of rapid sodium increase in patients with primary polydipsia when fluid restriction is enforced, check electrolytes every 2-6 hours

7. Central adrenal insufficiency

A: Hydrocortisone 100mg i.v., followed by 50mg i.v. every 6-8 hours according to stress level

B: Additionally fluid substitution (see 1.1) as needed

Treatment Goal: increase **P-Na ≥2 (max 10) mmol/l /24h**
→ if goal not reached: re-evaluate diagnosis / other treatment option
Overcorrection: ≥12 mmol/l/24h rsp. 18 mmol/l/48h
→ stop intervention, evaluate counter regulatory actions

Controls while hyponatremic:
<120 mmol/l: every 6-12 hrs
≥120 mmol/l: every 12-24 hrs

EXPANDED ECF

8. Kidney disease
A: Fluid restriction <1L/d
B: Limit maximum salt intake to 4g/d
C: Start or increase diuretics*:

- ***eGRF 30-45ml/min:*** Furosemide 3x 20-40mg i.v. (daily dose 60-120mg i.v.) or Torasemide 10-20 mg p.o
- ***eGRF 15-30ml/min:*** Fuosemide 3x 80-125mg i.v. (daily dose 250-375mg i.v.) or Torasemide 50-100mg p.o
- ***Acute oliguria:*** Furosemide up to 3x125-250mg i.v. (daily dose 375-750mg i.v.)

CAVE: adjust diuretic dosages according to comorbidities and co-medication

9. Nephrotic syndrome
A: Treatment of underlying disease
B: Diuretics*:

- ***Normal eGFR:*** Furosemide 3x 40-80 mg i.v. , increase up to 500mg i.v. as needed or Torasemide 40-60mg p.o, increase up to 200mg p.o. as needed
- ***Reduced eGFR:*** Furosemide 3x 80-125 mg i.v., increase up to 500mg i.v. as needed

C: Evaluate limitation maximum salt intake to 4 g/d
D: Evaluate additional administration of an ACE-inhibitior or Angiotensin-II-RA and/or Amilorid
CAVE: adjust diuretic dosages according to comorbidities and co-medication

10. Hypoalbuminemia (= malnutrition induced, not inflammation induced)
A: Diet counseling, additional protein intake (e.g. 1-2 High Protein Nutrition drinks/)
B: Evaluate additional measures:
- enteral or parenteral nutrition or
- iv Albumin-Substitution (1g/kg body weight for 2 days or Albumin 20g i.v. 1x/d)

11. Heart failure
A: Start or increase diuretics*:

- **Start:** Furosemide 20mg i.v. every 8h (GFR <30ml/min: 40mg i.v. every 8h), or Torasemide 20-40 mg p.o
- **Increase:** Furosemide 40-80mg i.v. every 8h, increase up to 3x240mg/d as needed or Torasemide 50-100 mg p.o. , increase up to 200mg/d as needed

CAVE: adjust diuretic dosages according to comorbidities and co-medication
B: Evaluate additional fluid restriction <1 L/d
C: Evaluate additional limitation maximal salt intake 4g/d

12. Chronic liver disease
CAVE: Only if normal kidney function, otherwise consider step 2.1
A: Fluid restriction <1 L/d and/or limitation salt intake max 4g/d
B: Start or increase Spironolactone (+100mg) up to 400mg/d
CAVE: regular control p-potassium/creatinine mandatory
C: Evaluate administration additional diuretics*:
Furosemide 40mg i.v. or Torasemide 10-20mg p.o
CAVE: adjust diuretic dosages according to comorbidities and co-medication
D: In case of renal failure, evaluate administration of albumin:
Albumin 1g/kg body weight for 2 days or Albumin 20g i.v. 1x/d

CAVE: Close monitoring of patient concerning possible signs of hepatic encephalopathy (HE) is mandatory. If HE is suspected, treatment intervention needs to be stopped and gastroenterologists have to be consulted for further management.

*Instead of furosemide / torasemide an **equivalent loop diuretic** in **equivalent dosage** may be given.
Medication may be given as **bolus** or as **continuous infusion**
